# A Rare Presentation of Infective Endocarditis Complicating Severe Aortic Valve Stenosis

**DOI:** 10.3390/jcdd13050220

**Published:** 2026-05-21

**Authors:** Cyrine Sghaier, Marielle Morissens, Pierre-Emmanuel Massart, Jose Castro Rodriguez, Georgiana Pintea Bentea

**Affiliations:** 1 Department of Cardiology, CHU Namur–Sainte Elisabeth, 5000 Namur, Belgium; 2 Department of Cardiology, CHU Brugmann, 1020 Brussels, Belgium

**Keywords:** aortic valve stenosis, watchful waiting, endocarditis, case report

## Abstract

Background: Although less frequently encountered, aortic valve stenosis is associated with complications separate from its hemodynamic burdens, such as infective endocarditis. Case Summary: We report the case of a 77-year-old female patient with regular cardiac follow-up in the setting of an asymptomatic severe aortic stenosis, who presented to the emergency department with signs and symptoms of sepsis and acute decompensated heart failure. Echocardiography revealed two vegetations attached to the tricuspid valve, an abscess of the anterior aortic ring, and a high-velocity ventricular septal defect. The patient was started on adequate antibiotic therapy. Surgical treatment in an urgent manner (within a few days) was decided by the Heart Team, in accordance with the ESC guidelines on the management of infective endocarditis. Whilst awaiting surgery, the patient presented with a sudden hemodynamic deterioration a few days after diagnosis, with cardiopulmonary arrest and subsequent death. Discussion: We hypothesize that the patient developed an infective endocarditis of the degenerated stenotic aortic valve with extension from left to right via a ventricular septal defect, the development of which was facilitated by the high trans-aortic valve gradient. Some reported cases describe a ventricular septal defect as a complication of native aortic valve endocarditis, though not all involve concomitant aortic stenosis. In conclusion, our case illustrates a very rare scenario of infective endocarditis complicating aortic stenosis with fulminant development. This case highlights a rare, albeit severe complication associated with aortic stenosis and therapeutic challenges in managing the dismal evolution of endocarditis in this setting.

## 1. Introduction

Although less frequently emphasized, aortic valve stenosis may be associated with risks beyond its hemodynamic consequences [1]. Severe aortic stenosis has been linked to modestly increased susceptibility to infective endocarditis compared with structurally normal valves, although the absolute risk remains lower than that reported in patients with significant aortic regurgitation, prosthetic valves, or previous endocarditis [2]. Epidemiological studies estimate the annual incidence of infective endocarditis in native valve disease at approximately 5 to 10 cases per 100,000 individuals [3]. In this context, advanced aortic stenosis has been proposed as a contributing factor to infective endocarditis, possibly related to valve degeneration, turbulent flow, and endothelial disruption. Nevertheless, reported associations vary between studies, and the overall risk attributable to severe native aortic stenosis appears relatively limited [3]. Some reports have noted a possible association between higher peak transvalvular gradients and infective endocarditis, while lower rates have been observed in older patients with heavily calcified valves [3]. The coexistence of endocarditis and aortic stenosis is clinically challenging, as these conditions may potentiate one another. Turbulent flow across a stenotic valve can accelerate vegetation formation and increase the risk of embolic events, while the inflammatory destruction induced by infection may exacerbate valvular obstruction or precipitate hemodynamic instability. Moreover, symptoms of early endocarditis, such as fatigue, exertional dyspnea, or chest discomfort, can easily be misattributed to the underlying stenosis, potentially delaying diagnosis and appropriate intervention.

When infective endocarditis develops in the context of aortic stenosis, several observational cohorts describe higher in-hospital mortality, increased rates of cardiogenic shock, and greater need for urgent surgical intervention in these patients compared with those with endocarditis on otherwise normal valves [4]. This heightened vulnerability reflects both the fragile hemodynamic equilibrium of severe aortic stenosis and the reduced physiological reserve available to withstand the systemic inflammatory burden of infection.

The present case underscores the poor prognosis associated with this combination. A patient was presented with previously asymptomatic but severe aortic stenosis who, upon developing infective endocarditis, experienced rapid clinical deterioration culminating in circulatory collapse despite timely diagnosis and the initiation of broad-spectrum antibiotics. 

## 2. Case Presentation

We report the case of a 77-year-old woman who presented to the emergency department with chest pain and New York Heart Association (NYHA) class III exertional dyspnea that had progressively worsened over the preceding week, accompanied by marked asthenia and diaphoresis. 

Her medical history was significant for asymptomatic severe aortic valve stenosis diagnosed four years earlier. Routine follow-up a few months before the index event included a transthoracic echocardiogram that demonstrated a long-standing stable peak aortic valve velocity of 4.5 m/s, consistent with severe aortic stenosis but without pulmonary hypertension, left ventricular dysfunction, or other alarming features. Additionally, the patient underwent an exercise stress test (80 W, 145 bpm), which elicited no symptoms and showed an appropriate blood pressure response. In the absence of exertional symptoms or markers of early decompensation, a conservative watchful-waiting strategy was chosen, in alignment with the European Society of Cardiology guidelines on valvular heart disease available at that time [1].

**Figure 1:**
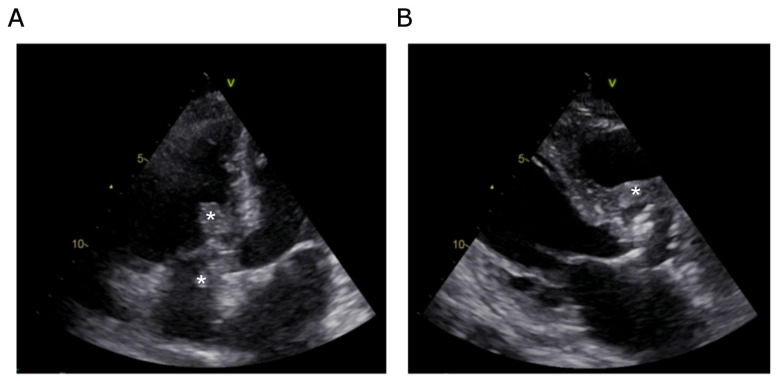
Two-dimensional transthoracic echocardiography images. (**A**) Apical view demonstrating two masses attached to the tricuspid valve, one on the atrial side and one on the ventricular side (asterisks). (**B**) Parasternal long-axis view showing thickening of the anterior aortic annulus, suggestive of abscess formation (asterisk). The aortic valve appears severely calcified, and the presence of a vegetation at this level cannot be excluded.

**Figure 2:**
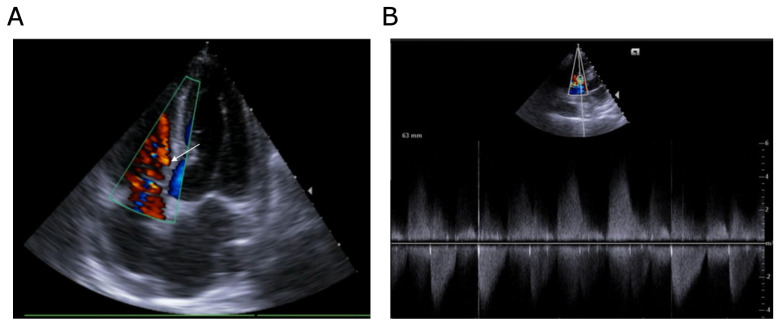
Two-dimensional transthoracic echocardiography images. (**A**) Apical view with color Doppler demonstrating a ventricular septal defect (arrow) in close proximity to the ventricular mass. (**B**) Parasternal short-axis view with continuous-wave Doppler showing a high-velocity jet across the ventricular septal defect (>5 m/s).

The patient’s presentation at the time suggested an acute cardiac process superimposed on underlying chronic valvular disease. However, upon arrival at the emergency department, the patient exhibited a low-grade fever (37.8 °C), tachycardia (110 bpm), normal blood pressure, and clinical signs of acute decompensated heart failure, including pulmonary crackles and peripheral congestion. A loud systolic murmur was immediately noticeable. Laboratory analyses revealed a systemic inflammatory response (C-reactive protein at 134 mg/L), markedly elevated cardiac biomarkers (troponin levels of 259 ng/L), and severe neurohormonal activation (NT proBNP levels of 26,317 ng/L). Given this constellation of findings, urgent cardiology evaluation was requested. The ECG showed sinus tachycardia with evidence of left ventricular hypertrophy, though without acute ischemic changes. The echocardiographic assessment proved pivotal. Transthoracic echocardiography identified two distinct mobile, friable, supracentimetric masses attached to the tricuspid valve—one on the atrial side and another on the ventricular side—both suspicious for vegetations (Figure 1A). Additionally, there was marked thickening of the anterior aortic annulus suggestive of abscess formation (Figure 1B). The aortic valve exhibited advanced calcification, rendering the presence of superimposed vegetations difficult to exclude. The aortic valve was severely stenotic, with a peak velocity of 4.5 m/s, a maximal gradient of approximately 80 mmHg, and a valve area of 0.7 cm^2^ calculated using the continuity equation. The right ventricle showed normal contractility, with no signs of pulmonary hypertension. A high-velocity ventricular septal defect (>5 m/s) was detected in close proximity to the ventricular mass (Figure 2), raising concerns about the presence of either infection-related septal destruction or a pre-existing defect exacerbated by inflammatory processes. Transesophageal echocardiography subsequently confirmed these abnormalities and reinforced the diagnosis of multivalvular infective endocarditis with suspected peri-annular extension.

The patient was promptly initiated on broad-spectrum intravenous antibiotics, including ceftriaxone, flucloxacillin, and gentamicin. All four blood cultures collected in the emergency department grew Streptococcus oralis within 24 h. The organism was penicillin-sensitive, prompting de-escalation of antibiotic therapy to penicillin GA. The patient met two major clinical criteria (positive blood cultures for a typical microorganism and echocardiographic evidence of vegetation and abscess), thereby fulfilling the modified Duke criteria for infective endocarditis [5]. Her case was discussed by the multidisciplinary Heart Team. Despite the severity of the infective process, her hemodynamic profile remained stable. Therefore, consistent with the European Society of Cardiology’s infective endocarditis guidelines [2], surgery was scheduled on an urgent basis (within several days) rather than an emergent (within 24 h) basis. This approach allowed for a short course of targeted antibiotic therapy to reduce the infectious burden in the hope of improving the operative outcome, given that surgery performed during uncontrolled infection is associated with significantly higher mortality [3].

The planned surgical approach included replacement of both the aortic and tricuspid valves. Intraoperative inspection would have enabled confirmation of the extent of peri-annular abscess formation, evaluation of the potential involvement of aorto-mitral continuity, and identification of any additional fistulous tracts: findings that frequently necessitate complex surgical reconstruction. Contemporary data also suggest that among elderly patients (>75 years), double valve surgery for infective endocarditis carries a substantial 90-day mortality exceeding 30%, highlighting the complexity and physiological strain associated with such procedures [4]. These considerations were weighed heavily in the Heart Team’s decision-making process. In addition, a repeat transthoracic echocardiogram performed after several days of antibiotics demonstrated stability of the endocarditis-related lesions, without evidence of further progression or complications.

Clinically, the patient initially showed signs of improvement, with partial resolution of inflammatory markers and symptomatic stabilization. Blood cultures became negative after 72 h of targeted antibiotic therapy. However, a few days after diagnosis and before the scheduled surgical intervention, the patients experienced an abrupt and profound hemodynamic collapse, leading to cardiopulmonary arrest and death despite resuscitative efforts.

Autopsy findings were striking and confirmed the clinical suspicions. Vegetations were present on both the aortic and tricuspid valves, accompanied by multiple abscess cavities involving the upper interventricular and interatrial septa. Several fistulous tracts were also identified, indicating extensive spread of the infection across cardiac structural planes. These findings underscore the fulminant nature of the disease process and explain the sudden hemodynamic deterioration.

## 3. Discussion

Infective endocarditis remains a serious condition, with contemporary in-hospital mortality rates approaching 24% [6]. The already considerable risk associated with infective endocarditis increases further when additional prognostic factors are present, such as advanced age, significant comorbidities, delays in diagnosis, persistent bacteremia, and the onset of clinical or echocardiographic complications such as abscesses, pseudoaneurysms, or conduction disturbances [7]. In the case presented here, several of these risk elements aligned, placing the patient in a highly vulnerable situation from the outset. Her sudden deterioration despite apparent early stabilization suggests that infective endocarditis often follows an unpredictable and potentially fulminant course, even under timely antimicrobial therapy.

Right-sided infective endocarditis represents a relatively uncommon entity, accounting for only 5–10% of endocarditis cases [8]. It is typically associated with a more favorable in-hospital prognosis: approximately 7% mortality compared with left-sided disease [8]. This difference reflects, in part, the distinct hemodynamic environment of the right heart, where lower pressures reduce the likelihood of large vegetations causing embolization to critical systemic organs. However, right-sided disease usually arises in clearly identifiable high-risk groups: patients with cardiac implantable electronic devices, individuals who inject drugs intravenously, and immunosuppressed patients [9]. The patient described here does not fall into any of these traditional categories. Instead, she presented with another important predisposing factor: severe aortic stenosis. This condition is increasingly recognized as a risk substrate for infective endocarditis [3]. The calcified, thickened, and irregular valve surface, combined with the extreme shear stress and turbulent flow generated by severe stenosis, disrupts endothelial integrity and facilitates microbial adhesion, thereby increasing susceptibility to infection. 

Structuring the clinical narrative provides a clearer view of the different aspects of the reported case.

Findings directly demonstrated by imaging

Transthoracic echocardiography showed two masses on the tricuspid valve (atrial and ventricular sides), suspicious for vegetations, along with anterior aortic annular thickening suggestive of abscesses. The aortic valve was heavily calcified with severe stenosis. A high-velocity ventricular septal defect (>5 m/s) was detected near the ventricular tricuspid mass. Transesophageal echocardiography confirmed these findings, consistent with multivalvular infective endocarditis and suspected peri-annular extension.

Clinically inferred mechanism

**Figure 3:**
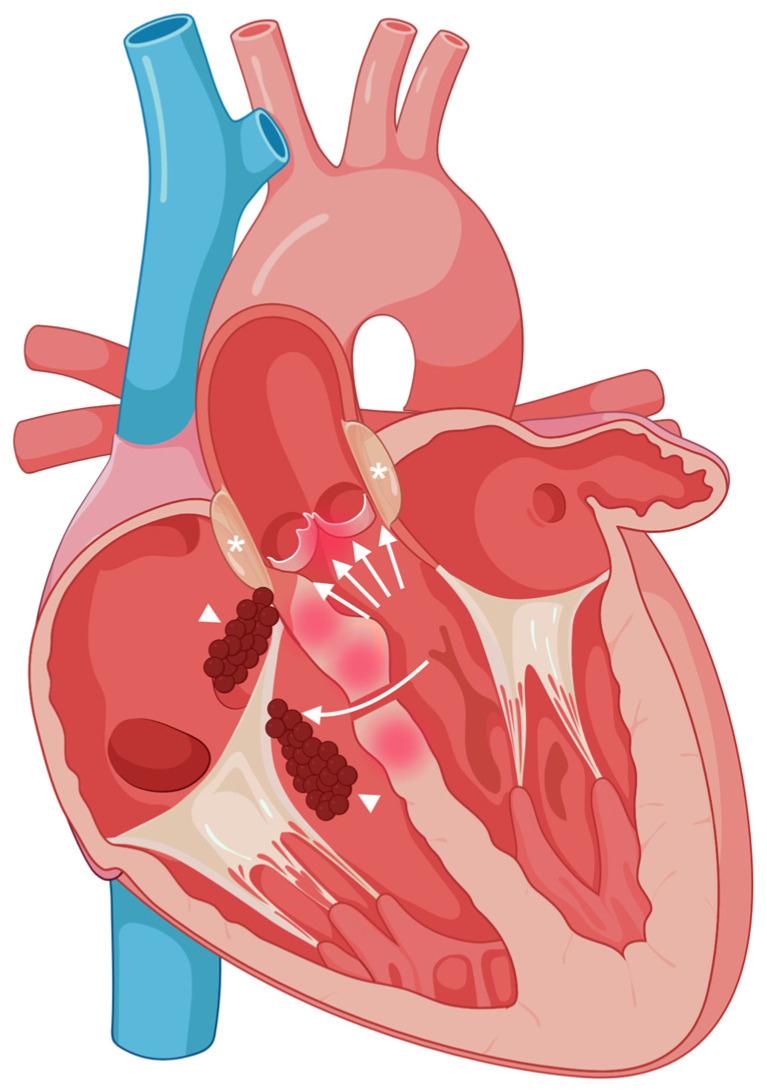
Schematic illustration of the proposed pathway of infective endocarditis extension. Asterisks denote the para-annular abscess of the aortic valve. The curved arrow indicates formation of a ventricular septal defect resulting from the combined contiguous spread of inflammation (highlighted in red) into the ventricular septum, and the elevated trans-aortic valve gradient (multiple arrows). Arrowheads identify vegetations on the tricuspid valve. Created in BioRender: https://BioRender.com/qboh1a7 (accessed on 10 May 2026).

We hypothesize that the infective process originated at the severely stenotic aortic valve, with subsequent extension from the left to right cardiac chambers through a ventricular septal defect. The markedly elevated transvalvular gradient characteristic of severe aortic stenosis likely amplified jet flow across the defect, creating conditions favorable for bacterial seeding on the right-sided structures: an uncommon but physiologically probable mechanism of dissemination. Moreover, the patient had undergone previous echocardiographic examinations as part of the follow-up for her aortic valve stenosis. Upon review of these images, a pre-existing ventricular septal defect was definitively excluded. A schematic diagram depicting the hypothesized route of infection spread is shown in Figure 3.

The patient’s abrupt hemodynamic collapse and death were most likely triggered by an acute increase in left-to-right shunt volume, superimposed on the high-gradient physiology. This sudden shift would have resulted in rapid right ventricular volume overload, progressive dilation, impaired contractility, and ultimately a critical reduction in systemic cardiac output. In this context, even brief delays in surgical management can rapidly lead to cardiovascular collapse, as occurred in this case.

Pathology confirmed at autopsy

Autopsy confirmed vegetations on both the aortic and tricuspid valves, along with multiple abscess cavities involving the upper interventricular and interatrial septa. Several fistulous tracts were identified, demonstrating extensive spread of the infection across cardiac structural planes.

There are some other learning points that are worth highlighting. 

Severe aortic stenosis is associated with a markedly adverse prognosis, even in the absence of overt symptoms. Although classical observations emphasize the precipitous decline in survival following symptom onset, contemporary data indicate that a substantial proportion of asymptomatic individuals already exhibit subclinical hemodynamic compromise [10]. This latent dysfunction contributes to an unfavorable natural history, underscoring the seriousness of the disease irrespective of symptom status.

Recent evidence challenges the traditional watchful-waiting approach in asymptomatic severe aortic stenosis. While intervention was historically deferred until symptom onset, newer data suggest substantial long-term mortality and early myocardial damage even in asymptomatic patients. Reflecting the accumulating evidence, the 2025 European Society of Cardiology Guidelines have expanded the indications for aortic valve interventions in patients with asymptomatic severe aortic stenosis [11]. This evolution is grounded in randomized trials and observational studies demonstrating superior clinical outcomes with early aortic valve replacement—whether surgical or transcatheter—compared with a conservative, watchful-waiting strategy [10,12]. The present case highlights a rare but potential complication that can occur during surveillance in patients with severe aortic stenosis, rather than supporting broader implications for routine management. 

Severe aortic stenosis increases susceptibility to infective endocarditis. While historically overshadowed by regurgitant lesions or prosthetic valves in terms of endocarditis risk, severe aortic stenosis nonetheless constitutes a clinically relevant substrate for native valve infection [3]. Moreover, in severe aortic stenosis, echocardiographic diagnosis of infective endocarditis is frequently challenging. Heavy valvular and annular calcification may generate acoustic shadowing, limiting visualization of vegetations, abscesses, and peri-annular extension, and reducing the sensitivity of transthoracic echocardiography. As a result, infection can be difficult to distinguish from advanced degenerative valve disease on imaging alone [13,14]. For this reason, diagnosis must rely on an integrated application of the modified Duke criteria. Persistent positive blood cultures for typical organisms remain a major criterion. In addition, new structural complications such as a ventricular septal defect—suggesting infective tissue destruction or fistulization—and clinical manifestations such as acute heart failure provide strong supportive evidence of infective endocarditis, particularly when echocardiographic visualization is limited [2].

The coexistence of stenosis and endocarditis results in a particularly deleterious hemodynamic interaction, as vegetations can aggravate the obstruction of an already critically narrowed valve, the high-velocity, high-shear-stress environment facilitates embolic phenomena, and the fixed outflow obstruction restricts the capacity for compensatory augmentation of cardiac output during systemic infection. The distinctive features of this case include the extensive septal destruction, the presence of a high-velocity shunt, and secondary tricuspid valve involvement in a patient lacking the classical risk factors for right-sided disease. Previous cases have reported ventricular septal defects as a complication of native aortic valve endocarditis [15,16,17]; however, not all of these involve aortic stenosis with endocarditis and an associated ventricular septal defect.

The optimal timing of surgery in endocarditis with severe aortic stenosis remains uncertain. There is limited scientific evidence guiding surgical timing when infective endocarditis arises on a severely stenotic valve. While early surgery is often recommended in complicated endocarditis, the presence of severe stenosis introduces additional considerations related to operative risk, tissue fragility, and hemodynamic instability. In retrospect, given this patient’s rapid deterioration, earlier intervention for either the stenosis or the endocarditis might have altered the clinical trajectory, particularly in the context of an associated ventricular septal defect.

This case describes a rare and severe presentation of infective endocarditis complicating severe aortic stenosis, with extension to right-sided structures through a ventricular septal defect, culminating in rapid circulatory collapse. It highlights a seldom-recognized yet severe complication of aortic stenosis and underscores the complex therapeutic challenges posed by endocarditis in this setting. Although infective endocarditis may occur in the setting of advanced valvular degeneration, the overall risk remains relatively low, and current evidence does not justify management strategies beyond established guideline-based care. Instead, this case highlights the importance of maintaining clinical awareness of infective endocarditis as a possible, albeit uncommon, complication in patients with severe native aortic stenosis, particularly when new systemic or infectious features are present.

## Data Availability

The data presented in this study are available on request from the corresponding author due to privacy reasons.
